# NURSES’ OPINIONS AND BELIEFS ABOUT MALNUTRITION IN OLDER ADULTS: A CROSS-SECTIONAL STUDY

**DOI:** 10.1093/geroni/igz038.1824

**Published:** 2019-11-08

**Authors:** Debbie Ten Cate, Roelof Ettema, Marieke J Schuurmans, Lisette Schoonhoven

**Affiliations:** 1 University of Applied Sciences Utrecht, Utrecht, Netherlands; 2 University Medical Center Utrecht, Utrecht, Netherlands

## Abstract

Malnutrition in older adults is a frequent and major problem. Despite the fact that nurses have an essential role in nutritional care, they fail to ensure appropriate delivery in preventing and treating malnutrition. For improvement, it is necessary to understand the perspective of nurses about malnutrition. The aim of this study was to gain insight into nurses’ opinions and beliefs about malnutrition in older adults. A cross-sectional study was conducted where nurses working in different health care settings were asked to fill in a survey with twelve questions regarding different aspects of malnutrition. Nurses (n = 557) frequently observe malnutrition in older care recipients, and they consider this as a serious health problem. They believe that prevention and treatment of malnutrition is important and they see screening of malnutrition as a relevant nursing activity. They also consider nutritional care as multidisciplinary. Nurses state their need for education to give adequate nutritional care. Nurses’ opinions and beliefs about malnutrition in older adults is positive, which enhances nurses’ behavior to give sufficient nutritional care to older adults. To gain more benefit in improving nursing activities within nutritional care for older adults, more education is needed targeting nurse professionals and nurse students.

